# Prevalence of Hepatitis D and Its Impact on the Clinical Efficacy of Antiretroviral Therapy in People With HBV/HIV-1 in Guangdong Province, China

**DOI:** 10.1093/ofid/ofaf764

**Published:** 2025-12-26

**Authors:** Yaozu He, Weiyin Lin, Hong Li, Fei Gu, Xianglong Lan, Xinhua Liu, Yeyang Zhang, RongHong Li, Ruiying He, Weiping Cai, Xiaoping Tang, Linghua Li

**Affiliations:** Infectious Disease Center, Guangzhou Eighth People's Hospital, Guangzhou Medical University, Guangzhou, China; Guangzhou Medical Research Institute of Infectious Diseases, Guangzhou, China; Infectious Disease Center, Guangzhou Eighth People's Hospital, Guangzhou Medical University, Guangzhou, China; Guangzhou Medical Research Institute of Infectious Diseases, Guangzhou, China; Infectious Disease Center, Guangzhou Eighth People's Hospital, Guangzhou Medical University, Guangzhou, China; Guangzhou Medical Research Institute of Infectious Diseases, Guangzhou, China; Infectious Disease Center, Guangzhou Eighth People's Hospital, Guangzhou Medical University, Guangzhou, China; Guangzhou Medical Research Institute of Infectious Diseases, Guangzhou, China; Infectious Disease Center, Guangzhou Eighth People's Hospital, Guangzhou Medical University, Guangzhou, China; Guangzhou Medical Research Institute of Infectious Diseases, Guangzhou, China; Infectious Disease Center, Guangzhou Eighth People's Hospital, Guangzhou Medical University, Guangzhou, China; Guangzhou Medical Research Institute of Infectious Diseases, Guangzhou, China; Infectious Disease Center, Guangzhou Eighth People's Hospital, Guangzhou Medical University, Guangzhou, China; Guangzhou Medical Research Institute of Infectious Diseases, Guangzhou, China; Infectious Disease Center, Guangzhou Eighth People's Hospital, Guangzhou Medical University, Guangzhou, China; Guangzhou Medical Research Institute of Infectious Diseases, Guangzhou, China; Institute of Infectious Disease, Guangzhou Eighth People's Hospital, Guangzhou Medical University, Guangzhou, China; Guangzhou Key Laboratory of Clinical Pathogen Research for Infectious Diseases Guangzhou, China; Infectious Disease Center, Guangzhou Eighth People's Hospital, Guangzhou Medical University, Guangzhou, China; Guangzhou Medical Research Institute of Infectious Diseases, Guangzhou, China; Infectious Disease Center, Guangzhou Eighth People's Hospital, Guangzhou Medical University, Guangzhou, China; Institute of Infectious Disease, Guangzhou Eighth People's Hospital, Guangzhou Medical University, Guangzhou, China; Infectious Disease Center, Guangzhou Eighth People's Hospital, Guangzhou Medical University, Guangzhou, China; Guangzhou Medical Research Institute of Infectious Diseases, Guangzhou, China

**Keywords:** antiretroviral therapy, clinical efficacy, HBV/HIV-1 coinfection, hepatitis D virus

## Abstract

**Background:**

Research on HDV prevalence among people with HBV/HIV coinfection in China is limited. The impact of HDV on antiretroviral therapy (ART) efficacy and liver disease progression in this population remains unclear.

**Methods:**

This retrospective cohort study included people with HBV/HIV-1 between 2005 and 2022. Baseline plasma was tested for HDV IgM/IgG; HDV RNA was measured if antibodies were positive. Demographics, liver complications, and ART responses were compared by HDV status.

**Results:**

Overall, 1130 people with HBV/HIV-1 were included, of whom 84 (7.4%) tested positive for HDV antibodies. Among these, 19 (22.6%) were HDV RNA-positive. Approximately 41.7% of HDV antibody-positive individuals had HCV coinfection. The median duration of ART was 7.4 years (interquartile range [IQR]: 5.1, 9.9). Longitudinal samples were available from 14 individuals with HDV RNA positivity. Baseline HDV RNA was 2.98 (IQR: 2.17, 4.78) log10 IU/mL. After a rapid decline during ART, 92.8% (13/14) of individuals reached undetectable levels at 7 years. When adjusted for HCV infection, HIV and HBV virological suppression, HBsAg clearance, and immunological nonresponders were comparable between HDV antibody-positive and -negative individuals (all *P* > .05), and between HDV RNA-positive and -negative individuals (all *P* > .05). The incidence rates of newly developed cirrhosis and hepatocellular carcinoma were also similar.

**Conclusions:**

HDV coinfection was observed in 7.4% of people with HBV/HIV-1, as a defective virus reliant on HBV, HDV RNA declined rapidly during long-term ART and HDV coinfection did not compromise HIV or HBV treatment efficacy.

Summary This study provides scientific justification by investigating HDV prevalence and its impact among people with HBV/HIV coinfection in China, offering insights into viral interactions and potential extensions for optimizing treatment strategies. These findings are significant for clinicians, researchers, and policymakers in China, as they demonstrate the negligible influence of HDV on ART efficacy and severe liver outcomes. Practically, physicians can focus on HIV and HBV suppression without routine HDV-specific interventions, thereby simplifying management for people with HBV/HIV coinfection in Chinese healthcare settings, while the spontaneous HDV clearance observed in most cases reinforces the adequacy of current ART protocols for this population.

Hepatitis D, also known as delta hepatitis, is a liver infection caused by the hepatitis D virus (HDV), a defective virus that requires the presence of the hepatitis B virus (HBV) for its replication [1]. HDV can accelerate liver damage, increase the risk of cirrhosis and hepatocellular carcinoma (HCC), and worsen overall prognosis [2, 3]. Globally, an estimated 5%–15% of individuals with chronic HBV infection are with HDV, although the prevalence can vary greatly depending on the geographic location and risk factors [4–7].

In the population with both HIV and HBV, the epidemiology of HDV is of particular concern. People with HIV (PWH) are at an increased risk of HDV infection primarily through shared parenteral routes, particularly intravenous drug use (IVDU), rather than sexual transmission. The prevalence of HDV among individuals with HBV/HIV can be significantly higher than those with HBV alone, with some studies showing that up to 21.4% of individuals with chronic HBV and HIV coinfection are also with HDV in high-risk regions [8–10]. In China, there are currently no specific national guidelines for hepatitis D, and HDV screening is not routinely recommended. In contrast, the 2024 WHO Guidelines recommend serological HDV testing for all HBsAg-positive individuals in settings with HDV prevalence >5% or in high-risk groups, with reflex HDV RNA testing for anti-HDV-positive cases [11]. Although the prevalence of HBV infection in China is high, research on the epidemiology of hepatitis D in populations with HBV/HIV is scarce. The impact of HDV coinfection on the efficacy of antiretroviral therapy (ART) and the progression of liver disease in individuals with HBV/HIV remain unclear.

Here, we conducted a large-scale (over 1000) retrospective cohort study of individuals with HBV/HIV, aiming to assess the epidemiological prevalence of HDV, describe its main characteristics, and evaluate its impact on clinical outcomes during ART.

## METHODS

### Participants

This retrospective study was conducted at Guangzhou Eighth People's Hospital (GEPH), affiliated to Guangzhou Medical University, Guangzhou, China. People were recruited through the National Free Antiretroviral Treatment Program between January 1, 2005 and August 31, 2022, as previously described [12, 13]. The criteria for inclusion were: (1) aged 18 years or older; (2) confirmed HIV infection at baseline through either anti-HIV antibody and HIV nucleic acid testing; (3) confirmed HBV infection at baseline through HBsAg assay; (4) ART-naïve at baseline; and (5) at least one liver imaging examination (eg, ultrasound or CT) within 1 year of the last follow-up. The exclusion criteria were as follows: (1) complications of acute viral hepatitis within 6 months of ART initiation, (2) follow-up duration of <12 months or follow-up visits occurring less frequently than once per year, (3) missing essential baseline information (eg, CD4+ T cell count, CD4+ T cell/CD8+ T cell ratio, and liver imaging), and (4) absence of stored plasma samples at baseline.

### Study Design

In this observational cohort study, the baseline visit was defined as the time of ART initiation. People were followed every 3 months until death, loss to follow-up, or December 31, 2023. Active anti-HBV regimens containing ART were initiated per standard care, with lamivudine (3TC) or emtricitabine (FTC) combined with tenofovir disoproxil fumarate (TDF) or tenofovir alafenamide (TAF) recommended as the first-line regimen. For people initiating ART before 2011, 3TC monotherapy was commonly used as the only HBV-active drug in their regimen, since TDF was not recommended as a first-line drug until the 2011 Chinese Guidelines for the Diagnosis and Treatment of HIV/AIDS [14]. These individuals were subsequently transitioned to combination regimens including 3TC or FTC with TDF or TAF after the guideline update, as part of routine clinical practice to optimize HBV suppression and minimize resistance. Liver, renal, and immune functions were routinely monitored, and HIV RNA, HBsAg, and HBV DNA levels were quantified annually. Liver ultrasonography findings and alpha-fetoprotein levels were monitored annually. Immunological nonresponders (INRs) were defined as individuals who received ART for more than 4 years [15], had a peripheral blood viral load below 50 copies/mL for over 3 years, and had a persistent CD4+ T cell count lower than 350/µL, without other potential causes of low CD4+ T cell counts, such as immune deficiencies, chronic viral infections, hematologic malignancies, or long-term use of immunosuppressive drugs. Serum samples collected before and after ART initiation were stored at −80°C for HBV DNA, HDV serology, and HDV RNA quantification.

### Laboratory Assessments

CD4+ T cell counts were analyzed using flow cytometry (BD Biosciences, San Jose, CA, USA). Plasma HIV RNA levels were quantified using the COBAS automated viral load analysis system (COBAS TapMan48; Roche, Basel, Switzerland), with a detection limit of 20 copies/mL. Plasma HBV DNA levels were measured using an HBV DNA assay kit (Da'an, Guangzhou, China) and quantified using a CFX96 Touch Real-Time PCR detection system (Bio-Rad, Hercules, CA, USA) with a lower detection limit of 100 IU/mL. HBV serological markers, including HBsAg, anti-HBs, HBeAg, anti-HBe, and HCV antibodies, were evaluated using a chemiluminescent enzyme immunoassay (MODULAR E601 system, Roche). HDV-IgM and HDV-IgG were detected using an HDV antibody test kit with an enzyme-linked immunosorbent assay (Wantai Biopharm, Beijing, China). Positivity was determined as follows: for HDV-IgM, a sample was considered positive if the absorbance value (OD) was greater than or equal to the cutoff value, calculated as the mean absorbance of the negative control plus 0.16 (as specified in the HDV-IgM test kit instructions); for HDV-IgG, a sample was deemed positive if the absorbance value (OD) was greater than or equal to the cutoff value, calculated as the mean absorbance of the negative control plus 0.16 (as specified in the HDV-IgG test kit instructions). HDV RNA was quantified using an HDV RNA 2.0 PCR kit (Roboscreen GmbH, Leipzig, Germany) with a lower detection limit of 6 IU/mL. The biochemical parameters of liver function were assessed using an automated biochemical analyzer (Roche). The normal ranges for alanine aminotransferase (ALT) and aspartate aminotransferase (AST) are 9–40 and 9–35 U/L, respectively. Liver fibrosis was assessed using the AST-to-platelet ratio index (APRI) and fibrosis-4 (FIB-4) scoring systems, calculated based on AST, platelet count, age, and ALT levels, with thresholds for staging fibrosis (eg, APRI < 0.5 for no/minimal fibrosis, >2.0 for cirrhosis; FIB-4 < 1.45 for no/minimal fibrosis, >3.25 for cirrhosis). Cirrhosis was diagnosed using liver ultrasound (B-ultrasound) to evaluate liver surface nodularity, parenchymal texture, and portal hypertension signs, with CT scans used as an alternative, assessing nodular liver contour and vascular abnormalities via contrast-enhanced imaging.

### Statistical Analysis

Statistical analysis was performed using the SPSS software (version 20.0; SPSS Inc., Chicago, IL, USA). Data are presented as medians with interquartile ranges (IQR) for continuous variables, following normality testing (eg, Shapiro–Wilk or Kolmogorov–Smirnov test) that indicated non-normal distribution, and as frequencies (%) for categorical variables. The Mann–Whitney nonparametric test was used to compare continuous variables, whereas categorical variables were analyzed using the χ^2^ test or Fisher–Freeman–Halton test. To assess the risk of new-onset cirrhosis across the subgroups, Kaplan–Meier survival curves were constructed using GraphPad Prism (version 9.5). Between-group differences were evaluated by calculating hazard ratios using Cox proportional hazards regression to quantify the risk of new-onset cirrhosis, with statistical significance assessed at *P* < .05. Statistical significance was defined as a two-tailed *P* value of .05.

## RESULTS

### Study Population

**Figure 1.**
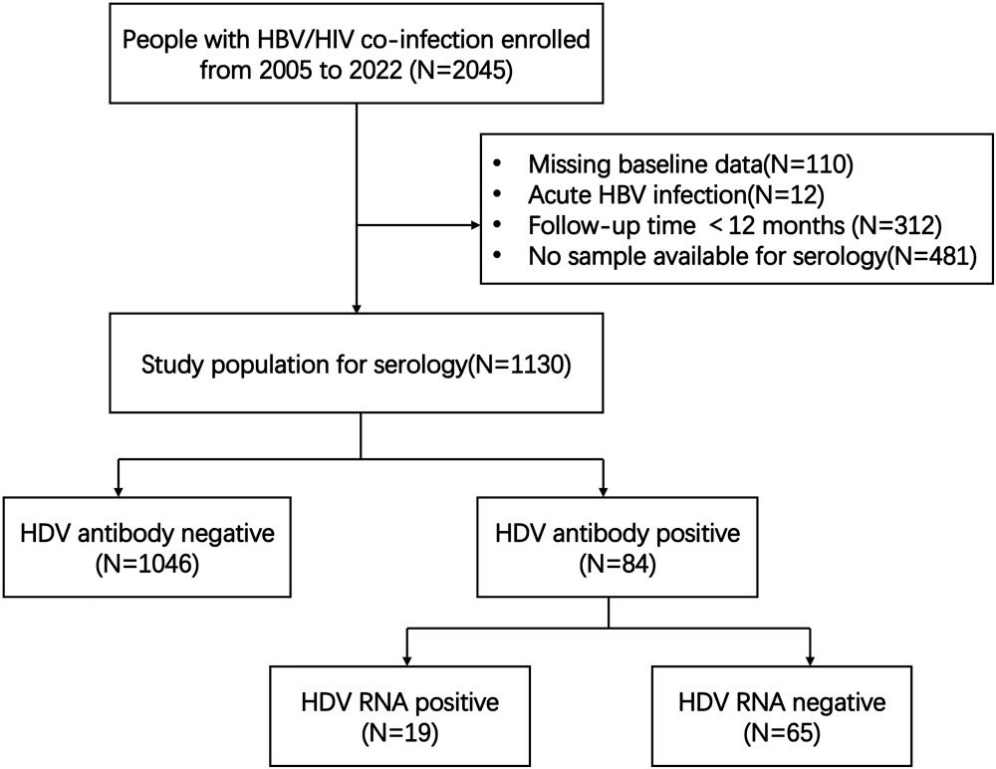
Flowchart of participants for analysis.

Overall, 2045 consecutive people with HBV/HIV-1 were enrolled between January 2005 and December 2022. Among these, 915 individuals were excluded for various reasons, including 110 with missing baseline data, 12 with acute HBV infection, and 312 with a follow-up period of less than 1 year due to referrals to other healthcare facilities, loss to follow-up, or death. Additionally, 481 individuals were excluded due to missing baseline samples (Figure 1). Ultimately, 1130 people with HBV and HIV-1 were included in the analysis.

### HDV Prevalence in People With HBV/HIV-1

Of the 1130 people with HBV/HIV-1, baseline samples were tested for HDV antibodies. Overall, 84 individuals (7.4%) tested positive for HDV antibodies: 29 with isolated IgM positivity, 20 with isolated IgG positivity, and 35 with both IgM and IgG positivity. Among the 84 HDV antibody-positive individuals, 19 (22.6%) had detectable HDV RNA levels with a median level of 3.43 (IQR: 1.99, 4.88) log10 IU/mL.

### Characteristics of HDV Infection

The baseline characteristics of the 84 individuals who tested HDV antibody-positive and 1046 individuals who tested antibody-negative are shown in Table 1. Compared to the antibody-negative group, the antibody-positive group was older, had a higher proportion of males, a greater prevalence of IVDU, a higher rate of HCV coinfection, a lower body mass index, and a lower CD4+ T cell/CD8+ T cell ratio (all *P*<.05). Additionally, the antibody-positive group had higher ALT and AST levels, lower platelet (PLT) counts, and higher noninvasive liver fibrosis indices (APRI and FIB-4) (all *P*<.05). No significant differences were found between the 2 groups in terms of WHO stage, HIV RNA levels, HBV DNA levels, HBeAg status, CD4+ T cell count, cirrhosis, and initial anti-HBV regimens (all *P* > .05).

**Table 1.** Baseline Characteristics of HBV/HIV Participants by HDV Ab Status

Characteristics	HDV Ab Positive *n* = 84	HDV Ab Negative *n* = 1046	*P* Values
Age, median (IQR)	37.1 (32.3, 45.1)	34.8 (28.5, 43.2)	.**029**
Gender, *n* (%)			.**026**
Male	77 (91.6%)	859 (82.1%)	
Female	7 (8.4%)	187 (17.9%)	
Transmission ways, *n* (%)			**<.001**
IDU	24 (28.6%)	27 (2.6%)	
Heterosexual	37 (44.0%)	518 (49.5%)	
Homosexual	19 (22.6%)	426 (40.7%)	
Sexual contact and IVDU	2 (2.4%)	4 (0.4%)	
Blood transfusion	1 (1.2%)	9 (0.9%)	
Others	1 (1.2%)	62 (5.9%)	
WHO stage, *n* (%)			.438
I	1 (1.2%)	30 (2.9%)	
II	1 (1.2%)	41 (3.9%)	
III	76 (90.5%)	890 (85.1%)	
IV	6 (7.1%)	85 (8.1%)	
BMI, median (IQR)	21.6 (19.8, 24.2)	20.8 (19.1, 23.2)	.**030**
HCV antibody, *n* (%)			**<.001**
Positive	35 (41.7%)	63 (6.0%)	
Negative	49 (58.3%)	983 (94%)	
HBV DNA in log10 IU/mL, median (IQR)	3.21 (2.43, 6.85)	4.64 (2.74, 7.24)	.100
HIV RNA in log10 copies/mL, median (IQR)	5.35 (4.7, 5.92)	5.37 (4.88, 5.77)	.585
CD4 count, cells/µL, median (IQR)	176 (57.0, 304.0)	218 (72.0, 340.5)	.135
CD8 count, cells/µL, median (IQR)	718 (418, 1291)	752 (498.3, 1073.8)	.620
CD4/CD8 ratio, median (IQR)	0.18 (0.09, 0.31)	0.25 (0.12, 0.41)	.**021**
ALT, U/L, median (IQR)	37 (27, 56)	29 (20, 46)	**<.001**
AST, U/L, median (IQR)	35 (27, 55)	27.7 (22, 41)	**<.001**
TBIL, µmol/L, median (IQR)	9.0 (6.6, 11.2)	8.8 (6.4, 12.2)	.754
PLT (10^9^/L), median (IQR)	172 (133, 218)	195 (156, 240)	.**013**
APRI, *n* (%)			.**002**
<0.5	37 (44.0%)	647 (61.8%)	
[0.5, 2.0]	42 (50.0%)	325 (31.1%)	
＞2.0	5 (6.0%)	74 (7.1%)	
FIB-4, *n* (%)			.**453**
<1.45	55 (65.5%)	752 (71.9%)	
[1.45, 3.25]	21 (25.0%)	210 (20.1%)	
＞3.25	8 (9.5%)	84 (8.0%)	
HBeAg state, *n* (%)	*n* = 76	*n* = 772	.611
Positive	21 (27.6%)	235 (30.4%)	
Negative	55 (72.3%)	537 (69.6%)	
Liver cirrhosis, *n* (%)			.447
Yes	10 (11.9%)	98 (9.4%)	
No	74 (88.1%)	948 (90.6%)	
Initial anti-HBV regimen, *n* (%)			.083
3TC	15 (17.8%)	120 (11.5%)	
3TC or FTC plus TDF or TAF	69 (82.2%)	926 (88.5)	

Data were shown as median (IQR) or *n* (%). The Mann–Whitney U test, as appropriate, was used to compare the quantitative variables, and the χ^2^ test or Fisher's exact test, as appropriate, was used to compare the qualitative variables between groups. Levels of significance: *P* = .05.

Abbreviations: Ab, antibody; ALT, alanine aminotransferase; APRI, aspartate aminotransferase to platelet ratio index; AST, aspartate aminotransferase; BMI, body mass index; FIB-4, fibrosis-4 score; FTC, emtricitabine; HBV, hepatitis B virus; HBeAg, hepatitis B e antigen; HCV, hepatitis C virus; HDV, hepatitis D virus; HIV, human immunodeficiency virus; IVDU, intravenous drug user; IQR, median; PLT, platelet; TAF, tenofovir alafenamide; TDF, tenofovir disoproxil fumarate; TBIL, total bilirubin; 3TC, lamivudine.

Among the 84 HDV antibody-positive individuals, we compared the baseline characteristics of 19 HDV RNA-positive individuals and 65 HDV RNA-negative ones (Table 2). The HDV RNA-positive group had a higher proportion of intravenous drug users, a higher rate of HCV coinfection, higher CD8+ T cell counts, elevated AST levels, lower PLT counts, and higher APRI and FIB-4 scores. No significant differences were observed in the other parameters.

**Table 2.** Baseline Characteristics of HBV/HIV Participants by HDV RNA Status

Characteristics	HDV RNA-Positive *n* = 19	HDV RNA-Negative *n* = 65	*P* Value
Age, median (IQR)	38.4 (32.5, 49.7)	37.0 (32.2, 44.8)	.477
Gender, *n* (%)			1.000
Male	17 (89.5%)	60 (92.3%)	
Female	2 (10.5%)	5 (7.7%)	
Transmission ways, *n* (%)			.**031**
IDU	8 (42.1%)	16 (24.6%)	
Heterosexual	8 (42.1%)	29 (44.6%)	
Homosexual	1 (5.3%)	18 (27.7%)	
Sexual contact and IVDU	0 (0%)	2 (3.1%)	
Blood transfusion	1 (5.3%)	0 (0%)	
Others	1 (5.2%)	0 (0%)	
WHO stage, *n* (%)			.860
I	0 (0%)	1 (1.5%)	
II	0 (0%)	1 (1.5%)	
III	18 (94.7%)	58 (89.2%)	
IV	1 (5.3%)	5 (7.8%)	
BMI, median (IQR)	21.5 (19.4, 23.3)	21.8 (19.9, 24.9)	.246
HCV antibody, *n* (%)			.**007**
Positive	13 (68.4%)	22 (33.8%)	
Negative	6 (31.6%)	43 (66.2%)	
HBV DNA in log10 IU/mL, median (IQR)	4.9 (3.4, 6.19)	3.0 (2.335, 6.9)	.222
HIV RNA in log10 copies/mL, median (IQR)	5.3 (4.8, 5.9)	5.4 (4.9, 5.9)	.994
CD4 count, cells/µL, median (IQR)	154.5 (26.5, 291.0)	185.0(60.0, 294.5)	.403
CD8 count, cells/µL, median (IQR)	558.0 (339.5, 814.0)	867.0 (522.5, 1339.5)	.**046**
CD4/CD8 ratio, median (IQR)	0.20 (0.07, 0.34)	0.18 (0.09, 0.30)	.617
ALT, U/L, median (IQR)	51.0 (32.6, 84.3)	36.3 (26.0, 50.0)	.090
AST, U/L, median (IQR)	51.0 (33.5, 66.9)	32.3 (25.5, 48.5)	.**043**
TBIL, µmol/L, median (IQR)	8.9 (7.6, 11.0)	9.1(6.6, 11.3)	.890
PLT (10^9^/L), median (IQR)	195 (141, 236)	195 (156, 241)	.646
APRI, *n* (%)			.**018**
<0.5	3 (15.8%)	34 (52.3%)	
[0.5, 2.0]	14 (73.7%)	28 (43.1%)	
＞2.0	2 (10.5%)	3 (4.6%)	
FIB-4, *n* (%)			.**017**
<1.45	7 (36.8%)	47 (72.3%)	
[1.45, 3.25]	8 (42.1%)	13 (20.0%)	
＞3.25	4 (21.1%)	5 (7.7%)	
HBeAg state, *n* (%)	*n* = 17	*n* = 59	1.000
Positive	5 (29.4%)	16 (27.1%)	
Negative	12 (70.6%)	43 (72.9%)	
Liver cirrhosis, *n* (%)			.848
Yes	3 (15.8%)	7 (10.8%)	
No	16 (84.2%)	58 (89.2%)	
Initial anti-HBV regimen, *n* (%)			.942
3TC	4 (21.1%)	11 (16.9%)	
3TC or FTC plus TDF or TAF	15 (78.8%)	54 (83.1%)	

### HDV RNA Dynamics in ART People

**Figure 2.**
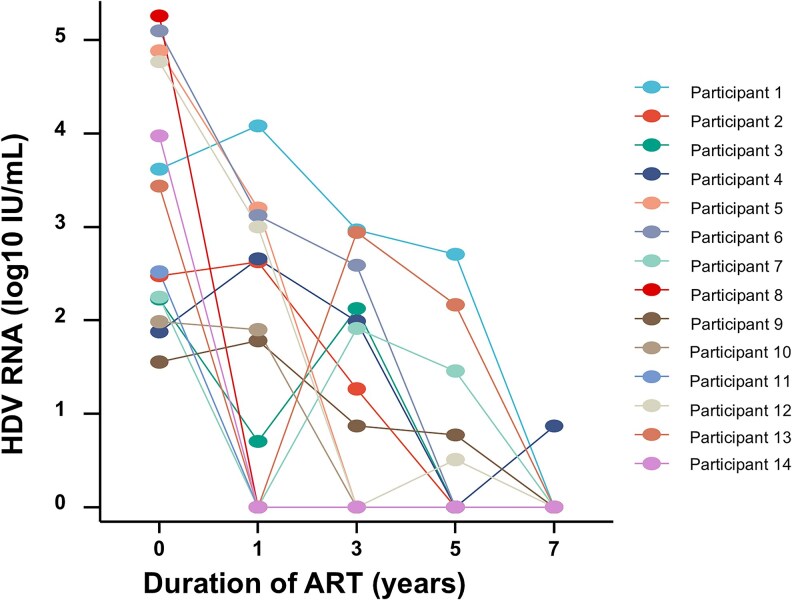
Changes in HDV RNA dynamics after ART.

Of the 19 baseline HDV RNA-positive individuals, serial specimens were retained from 14 participants. The baseline HDV RNA load of the 14 participants was 2.98 (IQR: 2.17, 4.78) log10 IU/mL. Regarding their ART regimens, 10 of the 14 participants (71.4%) were initially treated with an anti-HBV regimen containing TDF, while the remaining 4 participants were initially treated with an anti-HBV regimen consisting solely of 3TC and were subsequently switched to regimens including TDF. After ART, HDV RNA levels significantly decreased, as shown in Figure 2, with 92.5% (13/14) of the participants reaching undetectable levels at 7 years of ART.

### Impact of HDV Infection on ART Efficacy and Clinical Outcomes

**Figure 3.**
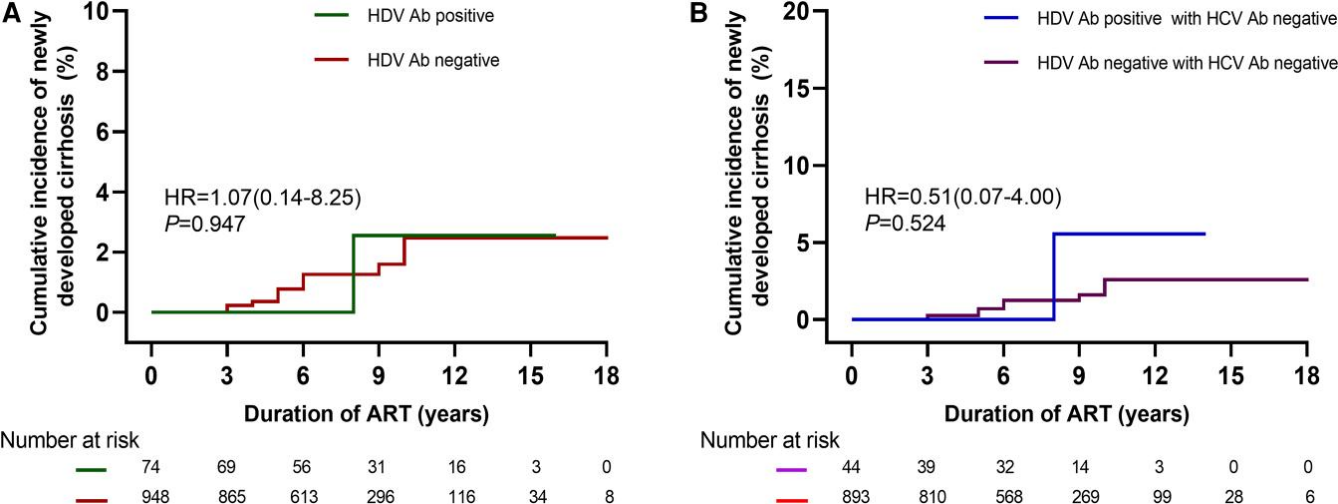
Cumulative incidence of newly developed liver cirrhosis by HDV status. *A*, Stratified by baseline HDV Ab status in the overall population. *B*, Stratified by baseline HDV Ab status among HCV Ab-negative participants.

The median follow-up time for HDV antibody-positive individuals was 8.3 years (IQR, 5.1–9.8), compared to 7.4 years (IQR, 6.0–11.0) for HDV antibody-negative individuals. At the last follow-up, 97.6% of the HDV antibody-positive group achieved HIV viral suppression, and all achieved HBV virological suppression, with no significant difference from the antibody-negative group (HIV suppression rate, 99.1%; HBV suppression rate, 98.6%; Table 3). Among the 74 participants who were HDV antibody-positive with more than 4 years of follow-up, 11 (14.7%) experienced INR, whereas 12.4% (110/948) of the antibody-negative participants experienced the same outcome, with no notable difference. Of the 74 HDV antibody-positive and 948 HDV antibody-negative participants without baseline liver cirrhosis, 1 (1.4%) and 13 (1.4%) developed liver cirrhosis, respectively; the 2 groups showed no significant difference (Figure 3*A*). In terms of HCC, 2 cases were observed in the HDV antibody-positive group compared to one in the antibody-negative group (2.4% vs 0.4%, *P* = 1.000). After adjusting for HCV coinfection, the 2 groups showed no differences in these outcomes (Supplementary Table 1).

**Table 3.** Treatment Response and Outcomes of HBV/HIV Participants by HDV Ab Status

Characteristics	HDV Ab Positive *n* = 84	HDV Ab Negative *n* = 1046	*P* Value
HBV DNA suppression, *n* (%)	*n* = 84	*n* = 1031	
	84 (100%)	1017 (98.6%)	.572
HBsAg seroclearance, *n* (%)	8 (9.5%)	52 (5.0%)	.124
HIV RNA suppression, *n* (%)	82 (97.6%)	1037 (99.1%)	.194
INR, *n* (%)	*n* = 74	*n* = 887	
	11 (14.7%)	110 (12.4%)	.578
Newly developed liver Cirrhosis, *n* (%)	*n* = 74	*n* = 948	
	1 (1.4%)	13 (1.4%)	1.000
HCC, *n* (%)	2 (2.4%)	4(0.4%)	0.067

Abbreviations: INR, immunological nonresponder; HCC, hepatocellular carcinoma; HBsAg, hepatitis B surface antigen.

Subgroup analysis based on HDV RNA status revealed no significant differences between the HDV RNA-positive and HDV RNA-negative groups in HIV suppression rate, HBV suppression rate, incidence of INR, new-onset cirrhosis, or HCC at the last follow-up (see Table 4 and Figure 3*B*). Specifically, the HIV suppression rates were 100% versus 96.9%, the HBV suppression rates were 100% versus 100%, the incidence of INR was 11.8% versus 15.5%, the new-onset cirrhosis rate was 0% versus 1.7%, and the HCC incidence was 0% versus 3.1% for the HDV RNA-positive and HDV RNA-negative groups, respectively (all *P* values > .05). After excluding the influence of HCV coinfection, similar findings were observed (Supplementary Table 2).

**Table 4.** Treatment Response and Outcomes of HBV/HIV Participants by HDV RNA Status

Characteristics	HDV RNA-Positive *n* = 19	HDV RNA-Negative *n* = 65	*P* Value
HBVDNA suppression, *n* (%)	19 (100%)	65 (100%)	NA
HBsAg seroclearance, *n* (%)	3 (15.8%)	7 (10.8%)	.848
HIV RNA suppression, *n* (%)	19 (100%)	63 (96.9%)	1.000
INR, *n* (%)	*n* = 17	*n* = 58	
	2 (11.8%)	9 (15.5%)	1.000
Newly developed liver cirrhosis, *n* (%)	*n* = 16	*n* = 58	
	0 (0%)	1 (1.7%)	1.000
HCC, *n* (%)	0 (0%)	2 (3.1%)	1.000

## DISCUSSION

In this retrospective study, we found that the HDV antibody positivity rate in the HBV/HIV population was 7.4%. Among the HDV antibody-positive individuals, 22.6% had detectable HDV RNA, indicating an active hepatitis D infection. This is similar to the results of a recent multicenter national study conducted by Wang et al [16] in China, in which the HDV antibody positivity rate in a population with HBV/HIV was 7.91%. Another retrospective cohort study from Taiwan included 534 individuals with HBV/HIV, with an HDV infection rate of 6.7% [17]. The above studies somewhat reflect the prevalence of HDV among individuals with HBV/HIV in China. The relatively low HDV antibody positivity rate observed in mainland China (7.4% in our cohort and 7.91% overall in Wang et al. may be largely explained by the low proportion of IDU in the transmission of HIV. IDU is a well-established major risk factor for HDV transmission, contributing to high HDV prevalence in many endemic regions. In our study, only 4.5% (51/1130) of the overall HBV/HIV coinfected cohort reported a history of IDU, reflecting the predominantly sexual transmission of HIV in Chinese HBV/HIV populations.

Reports indicate that a subset of PWH do not normalize CD4+ T cell count and function. These individuals are unable to fully reconstitute their immune systems. Consequently, they experience higher mortality and are at an increased risk of developing non-AIDS-related diseases compared with that of individuals who successfully restore their immune function. HBV and HIV can synergistically suppress the host immune system. For example, HIV infection leads to a decrease in CD4+ T cells, weakening immune control over HBV, whereas persistent replication of HBV exacerbates chronic inflammation by activating the toll-like receptor pathway, further impairing immune reconstitution [18]. HDV infection triggers a cascade of immune responses, resulting in sustained immune activation that may further compromise HIV immune reconstitution. Global multicenter studies, such as the ALLIANCE study, have indicated that in coinfected individuals, CD4+ T cell recovery remains suppressed even with potent ART, particularly in populations with a baseline CD4+ T cell count < 200/µL [19]. A similar phenomenon was observed in a previous study [20]. Thus far, research on immune reconstitution in individuals with HBV/HIV and HDV has been limited. Our findings indicate that regardless of HDV infection status, HIV RNA is effectively controlled in individuals with HBV/HIV receiving ART, and the incidence of INR is similar, suggesting that HDV infection does not affect immune reconstitution in people with HBV/HIV.

HIV infection impairs the host immune system, potentially attenuating immune responses to HDV, facilitating its replication, and thereby increasing the risk of severe liver injury, cirrhosis, and liver failure. Several studies have reported the effects of HDV infection on the progression of liver disease in individuals with HBV/HIV, with European cohorts demonstrating substantially increased risks of severe liver-related events, HCC, and mortality [21, 22]. In contrast, our study found no significant differences in post-ART cirrhosis or HCC incidence between HDV antibody-positive and -negative individuals, despite elevated baseline ALT, FIB-4, and APRI in the former. These contrasting outcomes likely stem from regional and genotypic variation, with HDV genotype 2—predominant in China [16] typically exhibiting lower virulence and reduced cirrhosis/HCC risk compared with genotypes 1 and 5 prevalent in Western and African settings [23–25]. Additionally, our median follow-up of 7.4 years may have been insufficient to detect late-onset events, especially given the low baseline cirrhosis rate (11.9%). With only 84 HDV antibody-positive individuals (7.4% prevalence, ∼half that of cited cohorts), statistical power was limited for rare outcomes. The absence of HDV genotyping precluded direct confirmation of genotype effects. Notably, both the study by Wang et al. and the present study showed a lower proportion of HDV RNA positivity among HDV antibody-positive people in China, with lower HDV RNA levels in our study. This suggests that genotype differences may lead to variations in viral characteristics and virulence, which could influence disease progression.

The dynamics of HDV RNA after ART remain controversial. The SHCS cohort study reported that among 21 individuals with HBV/HIV who had detectable HDV RNA were treated with TDF-based ART for a median of 4.9 years, HDV RNA levels declined slowly, with only 3 people (14.2%) achieving undetectable levels [26]. In contrast, a Spanish study involving 19 individuals treated with TDF-based ART found that, after a median of 54 months, HDV RNA levels decreased more rapidly, with 53% of individuals reaching levels below the detection threshold [27]. In our study, 92.8% (13/14) of individuals with longitudinal samples achieved undetectable HDV RNA at 7 years. However, this high suppression rate is most likely explained by the predominance of HDV genotype 2 in China, which is associated with lower baseline replication, as evidenced by only 22.6% of anti-HDV-positive people having detectable HDV RNA at baseline. HDV is a defective RNA virus that depends on HBV-derived large HBsAg particles (33–36 nm) for infectious virion assembly [28–31]. Although nearly all individuals achieved HBV DNA undetectability post-ART, we lack data on large versus small HBsAg dynamics, precluding confirmation of whether HBV suppression directly reduced HDV infectivity. Quantifying HBsAg particle subtypes post-ART is essential to elucidate HDV RNA decline mechanisms.

Our study had some limitations. The small HDV RNA-positive group (*n* = 19) limited the power to detect differences in therapy efficacy and liver outcomes. Given the very low prevalence of liver cirrhosis at baseline as well as the low incidence of cirrhosis and HCC during follow-up, comparisons between HDV RNA-positive and -negative participants should be interpreted with caution. Liver stiffness was assessed using the APRI score instead of FibroScan; knowing that APRI performs poorly in HDV infection this represents an important limitation and may have underestimated fibrosis severity. Additionally, systematic recording of alcohol use history was lacking in our dataset, preventing assessment of its potential confounding effect on APRI/FIB-4 scores and liver disease progression. HDV genotypes were not determined, precluding genotype-specific analysis. Finally, we did not measure the surface antigens of different particle sizes, and the relationship between their changes after ART and HDV RNA remains unclear.

Overall, our study provides preliminary insights into the prevalence of HDV among people with HBV/HIV coinfection in China and indicates that HDV does not influence the effectiveness of ART or increase the risk of severe liver events. Notably, in the context of effective HIV and HBV suppression, most HDV RNA-positive individuals spontaneously clear the virus. Given the low HDV antibody positivity and active infection rates, their impact on liver disease progression appears limited; however, larger cohorts with longer follow-up focused on HDV RNA-positive people are needed to validate these findings and explore optimal treatment strategies for this subgroup.

## Supplementary Material

ofaf764_Supplementary_Data

## Supplementary Data

Supplementary materials are available at *Open Forum Infectious Diseases* online. Consisting of data provided by the authors to benefit the reader, the posted materials are not copyedited and are the sole responsibility of the authors, so questions or comments should be addressed to the corresponding author.
