# Supplementary material for: Prevalence of Hepatitis D and Its Impact on the Clinical Efficacy of Antiretroviral Therapy in People With HBV/HIV-1 in Guangdong Province, China
Source: Open Forum Infect Dis. 2025 Dec 26;13(1):ofaf764. doi: 10.1093/ofid/ofaf764 (PMC12740716; doi:10.1093/ofid/ofaf764)
Supplement: ofaf764_Supplementary_Data [file ofaf764_supplementary_data.docx]

**Prevalence of hepatitis D virus infection and its impact on the clinical efficacy of antiretroviral therapy in patients co-infected with hepatitis B and HIV**

**CONTENTS**

**1. Supplementary Materials ...................................... 1**

**Supplementary Table S1: [Treatment response and outcomes of HBV/HIV co-infected participants excluding HCV infection by HDV Ab status. ] ................2**

**Supplementary Table S2: [Treatment response and outcomes of HBV/HIV co-infected participants excluding HCV infection by HDV RNA status.] .............2**

**Supplementary Table 1.** Treatment response and outcomes of HBV/HIV co-infected participants excluding HCV infection by HDV Ab status.

| Characteristics | HDV Ab positive  n=49 | HDV Ab negative  n=983 | *p value* |
| --- | --- | --- | --- |
| HBV DNA suppression, n (%) | n=49 | n=970 |  |
|  | 49（100%) | 958(98.8%) | 1.000 |
| HBsAg seroclearance , n (%) | 4(8.1%) | 48(4.9%) | 0.490 |
| HIVRNA suppression, n (%) | 47 (95.9%) | 975 (99.2%) | 0.078 |
| INR, n (%) | n=42 | n=822 |  |
|  | 6(14.3%) | 107 (13.0%) | 0.812 |
| Newly developed liver cirrhosis,  n (%) | n=44 | n=893 |  |
|  | 1 (2.3%) | 12 (1.3%) | 0.467 |
| HCC, n (%) | 1 (2.0%) | 3 (0.3%) | 0.177 |

Data were shown as median or n (%). The Chi-square test or Fisher’s exact test, as appropriate, was used to compare the qualitative variables between groups. Levels of significance: p = 0.05.

Abbreviations: Ab, antibody: HBV, hepatitis B virus; HDV, hepatitis D virus; HIV, human immunodeficiency virus; HCC, hepatocellular carcinoma; HBsAg, hepatitis B surface antigen; INR, immunological non-responder;

**Supplementary Table 2.** Treatment response and outcomes of HBV/HIV co-infected participants excluding HCV infection by HDV RNA status.

| Characteristics | HDV RNA positive  n=6 | HDV RNA negative  n=43 | p value |
| --- | --- | --- | --- |
| HBV DNA suppression, n (%) | 6 (100%) | 43(100%) | NA |
| HBsAg seroclearance , n (%) | 0(0%) | 4(9.3%) | 1.000 |
| HIV RNA suppression, n (%) | 6 (100%) | 41 (95.3%) | 1.000 |
| INR, n (%) | n=5 | n=37 |  |
|  | 0 (0%) | 6 (16.3%) | 0.770 |
| Newly developed liver cirrhosis,  n (%) | n=6 | n=38 |  |
|  | 0 (0%) | 1 (2.6%) | 1.000 |
| HCC, n (%) | 0 (0%) | 1( 2.3%) | 1.000 |
